# 

**Published:** 1970-10

**Authors:** 


### BRISTOL MEDICO-CHIRURGICAL JOURNAL

OCTOBER 1970

VOL. 85 (iv) No. 316

